# Different caveolin isoforms in the retina of melanoma malignum affected human eye

**Published:** 2007-06-15

**Authors:** Ágnes Ida Berta, Anna L Kiss, Ádám Kemény-Beke, Ákos Lukáts, Arnold Szabó, Ágoston Szél

**Affiliations:** 1 Department of Human Morphology and Developmental Biology, Semmelweis University Budapest, Hungary; 2 Department of Ophthalmology, University of Debrecen, Hungary

## Abstract

**Purpose:**

Caveolin-1 has been identified in Müller and pigment cells of rodents, but the distribution of caveolin isoforms has not been studied in the human retina. Since there are no relevant data in humans, we aimed to study the distribution of caveolins in the human retina.

**Methods:**

Our samples were derived from eyes affected by melanoma malignum choroideae that were enucleated. The distribution of the caveolins was examined by immunocytochemistry using isoform-specific antibodies.

**Results:**

In this study we report on the presence of different caveolin isoforms in many cell types of the human retina. These isoforms were present in several regions and layers in the human retina. Centro-peripheral changes have been detected: the distribution altered following the radier direction.

**Conclusions:**

This is the first demonstration of caveolin expression in the human retina. Our data suggest that caveolins play an important role in the function of retinal cells. Our observations refute previous assumptions that there is a shortage of caveolins in the retina. Since the retina contains a number of different neuronal and glial cell types, the caveolin expression of these cells can no longer be a matter of dispute.

## Introduction

Caveolins are such integral membrane proteins that are principal components of the special, ω-shaped plasma membrane invaginations called caveolae. Multiple forms of caveolin have been identified: caveolin-1-α, caveolin-1-β, caveolin-2, and caveolin-3. They differ in their specific properties and tissue distribution. Caveolin-1 and caveolin-2 may originate from a common ancestor. They are most abundantly expressed in adipocytes, endothelial cells, fibroblasts and smooth muscle cells [1-3]. The expression of caveolin-3 is thought to be muscle specific [4-6], although it has been shown that they are present in astroglial cells [7] and neurons of vegetative ganglions [8]. Scherer et al. [3] identified two caveolin isoforms in *Caenorhabditis elegans*. Their findings indicated that caveolins are structurally and fuctionally conserved across species from worms to human. These data suggest that caveolins might have an important evolutionary role. The short cytoplasmic domain of the N-terminal region of caveolin isoforms forms multivalent homo- and hetero-oligomers of caveolins [1]. In contrast to caveolin-1, caveolin-2 was not found to form homo-oligomers. This latter isoform exists mainly as a monomer [2], or it forms stable hetero-oligomeric complexes with caveolin-1 [3]. Thus, caveolin-2 may function as an accessory protein in conjunction with caveolin-1 [1]. It has been proposed that the caveolin family members function as scaffolding proteins to organize and concentrate specific lipids (cholesterol and glycosphingolipids), lipid-modified signaling molecules and G proteins within caveolae. Binding may suppress or inhibit enzyme activity through the caveolin scaffolding domain, which is a common caveolin domain.

There are only a few published reports about the presence and distribution of caveolin in the retina. Caveolin-1 was found to be present in the outer plexiform layer (OPL) of mouse retina in the synaptic ribbon in photoreceptor terminals [9]. In another study caveolin-1 was detected in various layers of the rat retina, from the inner plexiform layer (IPL) to the outer limiting membrane (OLM), suggesting that caveolin-1 is expressed in Müller cells. Using specific markers, Scherer et al. confirmed Müller cells do contain caveolin [10]. Caveolin-1 was also discovered to be present in pigment epithelial cells. Laser scanning confocal microscopical analysis of intact retinal pigment epithelium (RPE) localized caveolin-1 to the apical and basal surfaces [11]. Recently Kim et al. [12] reported, caveolin-1 was present in the majority of retinal layers in the rat retina. Caveolin-2 immunostaining was much weaker, however it was intensely detected around the blood vessels. Caveolin-2 stained in the processes of glial cells and Muller cells, but immunoreactivity was very limited in retinal neuronal cells including the ganglion cells, amacrine cells, bipolar cells, horizontal cells, and photoreceptor cells. Only central regions of the retina were involved in the study. No examinations were made about caveolin-3.

There are no data available about the distribution of caveolins in the human retina. Therefore, the aim of our study was to investigate the localization of caveolin isoforms in the human retina. Since immunocytochemical analysis of the retina requires special preparation, including immediate and precise fixation, we used only enucleated human eyes for our experiments. We chose retinas affected by melanoma malignum choroideae, a common reason for enucleation. The quantity and the distribution of the caveolin isoforms were obviously different in the human retina compared to other species. The caveolins were present in many regions and layers in the human retina. They were present in both neuronal and glial cell types, which suggests, that caveolins must play a role in the functions of these cells.

## Methods

### Material

Three patients were involved in the study: two males (ages: 50, 57) and one female (age: 50). These patients were operated after the diagnosis of posterior uveal melanoma (melanoma malignum choroideae) in 2005 at the Department of Ophthalmology, University of Debrecen. The enucleation was made without prior treatment (brachytherapy was disqualified). Thus the retinas were not damaged due to any other treatment and stayed as intact as possible.

After the enucleation the eyes were included into histopathological study. The histological types according to Callendar's classification were: spindle A (male patient, age: 50), dominantly spindle B (female patient, age: 50), and dominantly epitheloid tumor (male patient, age: 57), respectively. All experiments involving human subjects were carried out according to the Helsinki Declaration and with the approval of the Human Studies Ethical Committee, University of Debrecen.

### Preparation of the retina

Immediately after enucleation a dorsal stitch was made for orientation. A non-infiltrated part of the eye was removed for further investigation, the rest was sent to histopathology. The former section was subsequently placed in the fixative (4% paraformaldehyde, in 0.1 M phosphate buffer, pH 7.4), and incubated for 24 h at 4 °C. Thereafter the solution was replaced with 0.1 M phosphate buffered saline (PBS, pH 7.4), and rinsed for at least 24 h before further processing. The retina was carefully detached from the posterior eyecup. Extreme care was taken to obtain samples from the peripheral part of the retina, and from behind the ora serrata. These ciliary and iridic regions can not be separated from the ciliary body and the iris, so they were handled together. For frozen sections the retinas were incubated overnight in 30% sucrose (diluted in 0.1 M phosphate buffer), embedded in cryomatrix (ThermoShandon Pittsburgh, PA), and frozen in liquid nitrogen. Next, 10 μm thick radial sections were prepared on a ThermoShandon cryotome. For radial semithin sectioning three samples were taken from the human retina following a radial plane including the macular region (M), periphery (P), ciliary body (CB).

### Immunocytochemistry

Sections of 10 μm thickness were cut on a cryostat and dried onto poly-L-lysine coated glass microscope slides at 37 °C. Sections were then soaked in PBS for 20 min. A blocking solution of, 1% bovine serum albumin (BSA) with 0.1% Triton X-100 was applied for 2 h. The primary antibody was applied at 4 °C overnight. Primary antibodies anti-caveolin-1 (polyclonal rabbit IgG, Transduction Laboratories, BD Biosciences, San Jose, CA), 1:100, anti-caveolin-2 (monoclonal mouse IgG, Transduction Laboratories), 1:200, and anti-caveolin-3 (monoclonal mouse IgG, Transduction Laboratories) 1:100, were diluted in 1% BSA at 4 °C overnight. Caveolin-1 and -3 anti-rabbit and anti-mouse Alexa 488 (Molecular Probes, Invitrogen, Carlsbad, CA) were used as secondary antibodies. Since caveolin-2 gave a weakly detectable signal with the previous method, we used biotinylated anti-mouse IgG, then Streptavidin Alexa 488 (Molecular Probes), 1:100, to intensify the signal. Even with this extra method, caveolin-2 still gave a weak signal. For visualization of the cytoskeleton, we used Alexa fluor 594-labeled phalloidin (Molecular Probes) to stain F-actin (1:100). Vectashield HardSet Mounting Medium (Vector Laboratories, Burlingame, CA) with DAPI (4',6-diamidino-2-phenylindole) was used to cover the slides. When DAPI binds to DNA, its fluorescence is strongly enhanced, with excitation wavelength 350 nm (nuclei appear blue). Control reactions were made using rabbit and mouse normal serum, non-specific primary antibodies, and by omitting the primary antibodies to preclude non-specific binding. The retinas were inspected on a Zeiss Axiophot microscope Thornwood, NY, using the appropriate filter set for the fluorescent antibodies. Micrographs were obtained using an Olympus DP50 camera. Fluorescent double- or triple-labeled specimens were also inspected on a Radiance 2100 Multi Photon Imaging System coupled to a Nikon Eclipse E800 microscope using LaserSharp 2000. Both Adobe Photoshop 7.0 and Confocal Assistant were used for primary image processing.

## Results

### Caveolin-1

**Figure 1:**
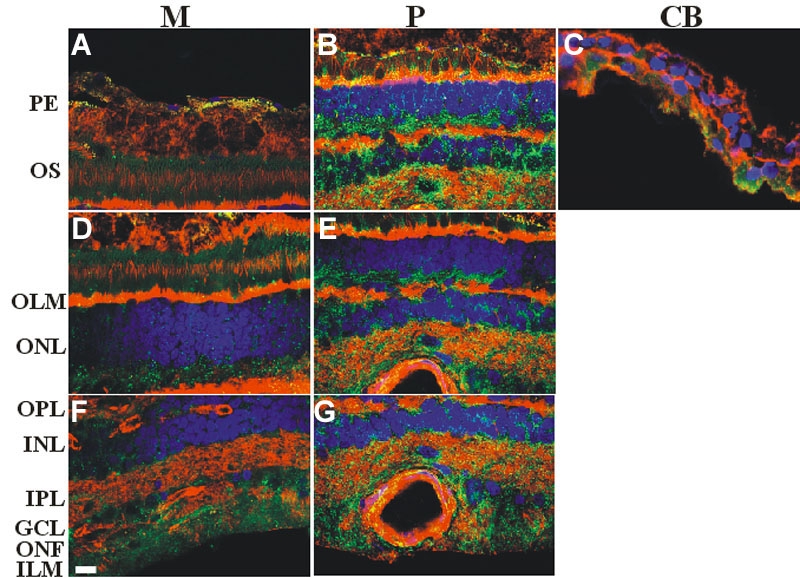
Immunocytochemical analysis of caveolin-1 in the human retina. Samples were taken from different sites the retina following a radial plane from the central to the peripheral retina. **A**, **D**, **F**: Macular region, M. **B**, **E**, **G**: Periphery, P. **C**: ciliary body, CB. Alexa Fluor 488 was used to detect caveolin-1 (green). The cytoskeleton and the nuclei were treated with Alexa fluor 594 labeled phalloidin (red) and DAPI (blue), respectively. Merged images are presented. Caveolin-1 was present in all layers of the retina. Weakest signals could be seen in the pigment epithelium (low **A**). The different regions showed a center-to-peripheral gradient; increasing from very low, low, moderate and high density (M, **A**, **D**, **F**), reaching the maximum level (very high; P, **B**, **E**, **G**), then decreasing towards the extreme periphery of the retina (very low, low; CB, **C**). Pigment granules showed autofluorescence, but they could be distinguished from caveolin signals. Scale bar represents 10 μm. The following abbreviations were used: pigment epithelium (PE), outer segments (OS), outer limiting membrane (OLM), outer nuclear layer (ONL), outer plexiform layer (OPL), inner nuclear layer (IPL), inner plexiform layer (INL), ganglion cells (GC), optic nerve fibers (ONF), inner limiting membrane (ILM).

The bound antibody was present in all layers of the retina. The weakest staining could be found in the pigment epithelium (low). The autofluorescence of the pigment granules made assessment of the immunostaining density more difficult. However the immunoreactive deposits were significantly smaller than the pigment granules, so the formers could be clearly identified. In other layers the density was estimated to vary from low to very high. The distribution of immunolabeling exhibited a characteristic center-to-peripheral gradient, increasing from very low, to low, moderate, and high density (M, Figure 1A,D,F), reaching the maximum level (very high; P Figure 1B,E,G), then decreasing towards the extreme peripheral part of the retina (very low, low; CB Figure 1C). (Figure 1, Table 1).

**Table 1:** Summary of labeling density of caveolin-1 at different sites in human retina.

**Region**	**M**	**P**	**CB**
PE	+	+++++	+
OS	++	+++++	++
OLM	++	+++++	
ONL	+++	+++++	
OPL	++++	+++++	
INL	+++	+++++	
IPL	+++	+++++	
GC	++++	+++++	
ONF	++++	+++++	
ILM	++++	+++++	

Caveolin-1 was present in all layers of the retina. Weakest signals were detected in the pigment epithelium (low). A center-to-peripheral gradient was observed: very low, low, moderate and high density at the central part (M), maximum level (very high) at the periphery (P), very low and low at ciliary body (CB). - indicates none; + indicates very low; ++ indicates low; +++ indicates moderate; ++++ indicates high; +++++ indicates very high.

### Caveolin-2

**Figure 2:**
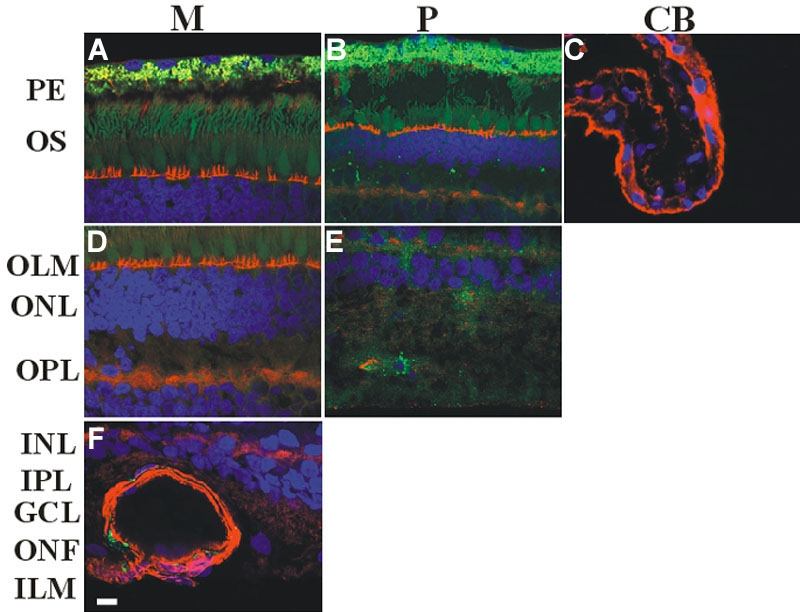
Immunocytochemical analysis of caveolin-2 in the human retina. Samples were taken as described in Figure 1. Alexa Fluor 488 was used to detect caveolin-2 (green). The cytoskeleton and the nuclei were treated with Alexa fluor 594 labeled phalloidin (red) and DAPI (blue), respectively. Merged images are shown. Caveolin-2 was found in the outer nuclear layer (ONL) and the inner limiting membrane (ILM), including the intervening layers. **A**, **D**, and **F** reveal: very low, low, and moderate densities (M). **B**, **E**: At the peripheral part (P) immunoreactivities (IRs) were observed only in the outer nuclear layer (ONL), outer plexiform layer (OPL), inner nuclear layer (INL), and ganglion cell (GC; very low, low). **C**: No signals were observed at the epithelium of the ciliary body (CB). Note, that pigment granules show autofluorescence. Scale bar represents 10 μm. The following abbreviations used in this figure: pigment epithelium (PE), outer segments (OS), outer limiting membrane (OLM), inner nuclear layer (IPL), optic nerve fibers (ONF).

Caveolin-2 immunoreactivities (IRs) were found to be present from the outer nuclear layer (ONL) to the inner limiting membrane (ILM), showing very low, low and moderate densities (M, Figure 2A,D,F). However no IRs were detected at the peripheral part (P, Figure 2B,E) in the IPL, the layer of the optic nerve fibers and the ILM. In the epithelium of the ciliary body (CB, Figure 2C) no IRs could be seen. (Figure 2, Table 2).

**Table 2:** Summary of labeling density of caveolin-2 at different regions in human retina.

**Region**	**M**	**P**	**CB**
PE	-	-	-
OS	-	-	-
OLM	-	-	
ONL	++	+	
OPL	++	++	
INL	+	++	
IPL	+	-	
GC	++	++	
ONF	+++	-	
ILM	+++	-	

Caveolin-2 was found in the outer nuclear layer (ONL) and the inner limiting membrane (ILM), including the intervening layers, showing very low, low and moderate densities (M). Very low and low density was seen at the periphery (P), but no signals were observed at the most peripheral part (CB). - indicates none; + indicates very low; ++ indicates low; +++ indicates moderate; ++++ indicates high; +++++ indicates very high. Following abbreviations are used: pigment epithelium (PE), outer segments (OS), outer limiting membrane (OLM), outer plexiform layer (OPL), inner nuclear layer (IPL), inner plexiform layer (INL), ganglion cells (GC), optic nerve fibers (ONF).

### Caveolin-3

**Figure 3:**
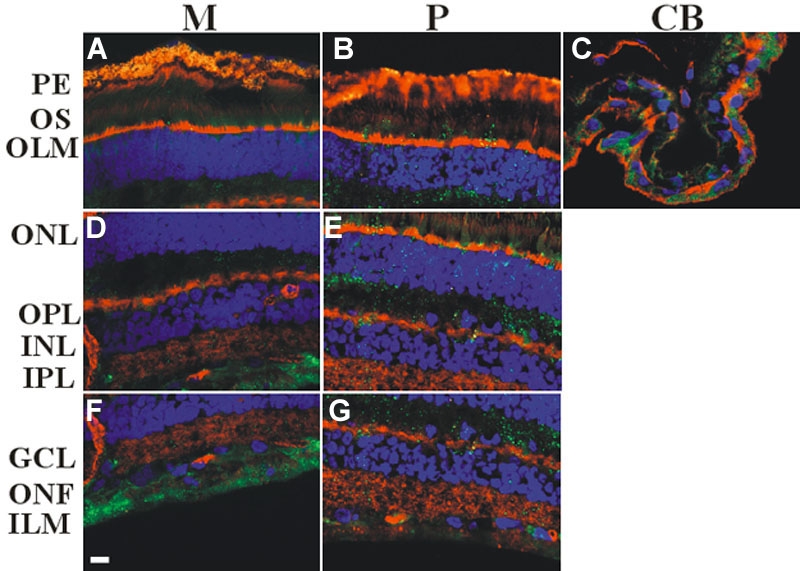
Immunocytochemical analysis of caveolin-3 in the human retina. Samples were taken as described in Figure 1. Alexa Fluor 488 was used to detect caveolin-3 (green). The cytoskeleton and the nuclei were treated with Alexa fluor 594 labeled phalloidin (red) and DAPI (blue), respectively. Presented images are merged. Caveolin-3 was observed in the outer nuclear layer (ONL) and the inner limiting membrane (ILM), including the intervening layers. **A**, **D**, and **F**: show very low, low, and moderate densities (M). **B**, **E**, **G**: At the peripheral part (P) IRs also occurred in the layer of the outer segments (low). **C**: At the ciliary body (CB), caveolin-3 signals could be seen in both layers (low, moderate). Note, that pigment granules show autofluorescence. Scale bar represents 10 μm. Following abbreviations are used in this figure: outer segments (OS), outer limiting membrane (OLM), outer plexiform layer (OPL), inner nuclear layer (IPL), inner plexiform layer (INL), ganglion cells (GC), optic nerve fibers (ONF).

In the macular region, caveolin-3 immunoreactivity was present from the INL to the ILM with very low, low and moderate densities (M, Figure 3A,D,F). At the periphery (P, Figure 3B,E,G) IRs also were in the layer of the outer segments (low). Caveolin-3 signals of low and moderate densities were present in both layers of the ciliary body (CB, Figure 3C, Table 3).

**Table 3:** Summary of labeling density of caveolin-3 in different locations of the human retina.

**Region**	**M**	**P**	**CB**
PE	-	-	++
OS	-	++	+++
OLM	-	-	
ONL	++	++	
OPL	++	++	
INL	+	++	
IPL	+++	++	
GC	++	++	
ONF	+++	++	
ILM	+++	++	

Caveolin-3 was observed in the outer nuclear layer (ONL) and the inner limiting membrane (ILM), including the intervening layers, showing very low, low and moderate densities (M). At the peripheral part (P) IRs immunoreactivities (IRs) also occurred in the layer of the outer segments (P). At the ciliary body (CB) caveolin-3 signals could be seen in both layers with low and moderate densities. - indicates none; + indicates very low; ++ indicates low; +++ indicates moderate; ++++ indicates high; +++++ indicates very high. Following abbreviations are used: pigment epithelium (PE), outer segments (OS), outer limiting membrane (OLM), outer plexiform layer (OPL), inner nuclear layer (IPL), inner plexiform layer (INL), ganglion cells (GC), optic nerve fibers (ONF).

Table 1, Table 2, and Table 3 present a summary of immunostaining densities in various tissue samples. Differences among the various sites were obvious, and only semiquantitative scoring was used. In contrast to monoclonal antibodies polyclonal sera may exhibit a reactivity against multiple epitopes. Accordingly, to avoid any uncertainty, caveolin isoform immunocytochemistry was performed with utmost care using a wide range of controls. For the same reason, the distribution of caveolin-1 detected by a polyclonal antibody was studied separately from that of the other two isoforms.

## Discussion

### Caveolin-1

Caveolin-1 expression has only been studied in rodent retinas. It was found to be present in the outer plexiform layer in mouse retina at the synaptic ribbon in photoreceptor terminals [9], and also detected in Müller cells and pigment epithelial cells of the rat retina [10,11]. Song et al. reported, caveolin-1 was present in the majority of retinal layers in the rat retina [12]. Caveolin-1 is known to be expressed abundantly in adipocytes, endothelial cells, fibroblasts, and smooth muscle cells. The observations of Song et al. and others confirm, that caveolin-1 is also expressed in some neuronal and glial cell types. No data are available about the distribution of caveolin-1 in the human retina yet, but studies on mammalian retinas suggest, that the densities of different caveolin types seem to be much lower compared to our sections. Caveolin-1 was only observed in a few cell types [9-11] and layers [12], including the ganglion cell layer, IPL, OPL, and in the vascular endothelial cells of the rat retina. Our observations show, caveolin-1 is evenly distributed among the different layers of the human retina, including the epithelium of the ciliary body. Although each cell type was not identified with specific markers, it was still obvious, that caveolin-1 is expressed in many cell types, including pigment cells, outer segments of photoreceptors, ganglion cells, and probably also in all the other cell types as well. Interestingly, the density of caveolin-1 exhibited a characteristic center-to-peripheral variation; after an increase toward the mid-periphery it reached a maximum level and then started to decrease toward the ora serrata.

### Caveolin-2

In previous studies, caveolin-2 was described to be present only around blood vessels of the rat retina [10,12]. This correlates with the fact, that caveolin-2 is expressed in adipocytes, endothelial cells, fibroblasts, and smooth muscle cells. Our observations confirmed the following: caveolin-2 is clearly localized around the human blood vessels. Caveolin-2 is also expressed in many layers in the human retina from the ONL to the ILM, indicating its presence in neuronal cell types. It is worth mentioning, that the most peripheral part of the human retina also expressed caveolin-2. The latter part, the epithelium of the ciliary body, was not examined previously in rodents.

### Caveolin-3

Until the present study, no studies examined the presence of caveolin-3 in the human retina. Caveolin-3 remains to be studied in rodent retinas. Traditionally, caveolin-3 has been thought to be muscle specific, however it has been detected in astroglial cells and vegetative ganglions [8]. It is also known that the expression of caveolin-3 is intense in the central nervous system in early embryonic stages in the chicken [13].

The distribution of caveolin-3 in the human retina was similar to caveolin-2: IRs were present from the ONL to the ILM. At the most peripheral regions, IR density was prominent. During retinal development, central portions of the retina develop first, and peripheral areas develop later [14-16]. Supporting previous observations about chicken development [13], caveolin-3 gives the highest density in this special, two layered peripheral part, which represents an early developmental stage of the retina.

### Conclusion

The quantity and distribution of the caveolin isoforms in the human retina are different from that found in other animals. Caveolins are present in more regions and in more layers in the human retina than in other species. Although caveolin is a conservative protein, the amount and distribution of caveolin in different tissues may exhibit considerable changes across species. Further data are needed to map the species-specific distribution of the detectable isoforms.

Further investigations are needed to explain the role played by caveolins in the intact human retina and in that of other species. Our observations demonstrate that in contrast to previous beliefs, there is no shortage of caveolins in the retina. It is hoped this study will facilitate additional research into the tissue specificity of caveolins. Since the retina contains a number of different neuronal and glial cell types, the caveolin expression of these cells can no longer be a matter of dispute.
